# Smart Water Management Platform: IoT-Based Precision Irrigation for Agriculture †

**DOI:** 10.3390/s19020276

**Published:** 2019-01-11

**Authors:** Carlos Kamienski, Juha-Pekka Soininen, Markus Taumberger, Ramide Dantas, Attilio Toscano, Tullio Salmon Cinotti, Rodrigo Filev Maia, André Torre Neto

**Affiliations:** 1 Center of Mathematics, Computing and Cognition, Federal University of the ABC, Santo André 09210-580, Brazil; 2 VTT Technical Research Centre of Finland, FI-90571 Oulu, Finland; Juha-Pekka.Soininen@vtt.fi (J.-P.S.); Markus.Taumberger@vtt.fi (M.T.); 3 Federal University of Pernambuco & Federal Institute of Pernambuco, Recife 50670-901, Brazil; rasd@cin.ufpe.br; 4 Department of Agricultural and Food Sciences (DISTAL), University of Bologna, 40127 Bologna, Italy; attilio.toscano@unibo.it; 5 Advanced Research Center on Electronic Systems “Ercole De Castro” (ARCES), University of Bologna, 40127 Bologna, Italy; tullio.salmoncinotti@unibo.it; 6 Centro Universitário FEI, São Bernardo do Campo 09850-901, Brazil; rfilev@fei.edu.br; 7 Brazilian Agricultural Research Corporation (EMBRAPA), São Carlos 13560-970, Brazil; andre.torre@embrapa.br

**Keywords:** Internet of Things, smart water management, smart agriculture, precision irrigation, IoT platform, FIWARE, linked data

## Abstract

The smart management of freshwater for precision irrigation in agriculture is essential for increasing crop yield and decreasing costs, while contributing to environmental sustainability. The intense use of technologies offers a means for providing the exact amount of water needed by plants. The Internet of Things (IoT) is the natural choice for smart water management applications, even though the integration of different technologies required for making it work seamlessly in practice is still not fully accomplished. The SWAMP project develops an IoT-based smart water management platform for precision irrigation in agriculture with a hands-on approach based on four pilots in Brazil and Europe. This paper presents the SWAMP architecture, platform, and system deployments that highlight the replicability of the platform, and, as scalability is a major concern for IoT applications, it includes a performance analysis of FIWARE components used in the Platform. Results show that it is able to provide adequate performance for the SWAMP pilots, but requires specially designed configurations and the re-engineering of some components to provide higher scalability using less computational resources.

## 1. Introduction

Agriculture is the biggest consumer of freshwater in the world, amounting to up to 70% of the total use [1], which makes the case for smart water management in order to guarantee water and food security to the world’s population. Irrigation systems and field application methods for the cultivation of crops play an important role therein. In an attempt to avoid loss of productivity caused by water stress (under-irrigation), farmers spray more water than needed (over-irrigation) and as a result not only productivity is challenged but also water and energy are wasted. Precision irrigation, in its turn, can use water more efficiently and effectively, avoiding both under-irrigation and over-irrigation. The smart management of water for precision irrigation in agriculture is essential for increasing crop yield and decreasing costs, while at the same time contributing to the environmental sustainability.

The Internet of Things (IoT) [2] emerges as the natural choice for smart water management applications, even though the integration of different technologies required for making it work seamlessly in practice is still not fully accomplished. The emergence of IoT is a phenomenon that owes to the conjunction of several factors such as inexpensive devices, low-power wireless technologies, availability of cloud data centers for storage and processing, management frameworks for dealing with unstructured data from social networks, high-performance computing resources in commodity platforms, and computational intelligence algorithms to deal with this monumental amount of data (aka big data analytics).

Currently, there are some challenges to be overcome that still prevent the widespread use of IoT for precision irrigation. Firstly, software development for IoT-based smart applications, such as irrigation for agriculture, is not yet fully automatized [3]. Secondly, advanced IoT software platforms are still missing, for automating part of the process and integrating different technologies such as IoT, big data analytics, cloud computing and fog computing, for the deployment of pilot applications for smart water management. Thirdly, the integration of heterogeneous and advanced sensors requires adequate standards and information models.

The SWAMP project developed and assessed an IoT-based smart water management platform for precision irrigation in agriculture with a hands-on approach based on four pilots in Brazil, Italy and Spain [4]. The SWAMP Platform can be configured and deployed in different ways thus making up different SWAMP Systems, customized to deal with the requirements and constraints of different settings, countries, climate, soils, and crops, which requires a good deal of flexibility to adapt to a range of deployment configurations involving a varied mix of technologies.

This paper presents the SWAMP project, its architecture, platform and pilots, as well as a scenario-based development process of derived systems. The SWAMP layered architecture considers three categories of services to ensure its replication and adaptability. Entirely replicable services deal with IoT services, storage services, and data analytics and machine learning. Fully customizable services deal with water data management issues that specialize generic analytic services into particular techniques for different types of irrigation and water distribution. Finally, application specific services require higher development effort since they serve particular farms.

The SWAMP Platform contains the mainstream components of FIWARE [5] and semantic features provided by a SPARQL-based context engine [6]. The platform may be deployed in a range of different configurations for component placement in the cloud or in the fog, involving the use of IoT communication technologies and smart algorithms and analytics in the cloud, and fog-based smart decisions located on the farm premises. This is aimed at experimenting with different deployment possibilities of the SWAMP Platform and providing additional insights in terms of the replicability and adaptability of its components to different settings. In other words, experimenting different deployment configurations is an important step for speeding up the learning process on how to deal with such a platform.

As scalability is a major concern for IoT applications, a performance analysis of key software components of its FIWARE-powered platform was conducted, personalized for each pilot scenario. The results show that this platform can deal with the requirements of the pilots, but scalability comes at a price. It was found that some FIWARE components must be fine-tuned to provide improved performance and other ones must be completely re-engineered to provide higher scalability using less computational resources. In addition, MongoDB was identified as the bottleneck of the FIWARE tested installation that may cause system crashes.

The contributions of this paper are threefold. Firstly, the SWAMP approach that is the result of a collaborative interdisciplinary project involving researchers and practitioners in two continents is introduced. Secondly, different system deployments for the SWAMP Platform are presented. Thirdly, the results of a performance analysis study that reveals important findings for the scalability of SWAMP IoT Platform based on FIWARE are shown.

In the remainder of this paper, Section 2 introduces background and related work. Section 3 introduces SWAMP concepts, while Section 4 presents the four pilots and Section 5 details the system deployment scenarios for the SWAMP Platform. Section 6 presents performance analysis results focusing on scalability of the SWAMP Platform. Section 7 discusses the key findings and results and finally Section 8 draws conclusions and future work.

## 2. Background and Related Work

To the best of our knowledge, there is no open IoT-based Platform specifically focused on precision irrigation for agriculture, so that a clear comparison with SWAMP is not possible. On the other hand, IoT has many security requirements, such as privacy, confidentiality, integrity, authentication, authorization and accounting [7], as well as the significant challenges posed by security threats to the success of IoT platforms [8]. However, this is out of the scope of this paper.

### 2.1. IoT Systems for Precision Irrigation

Current open source IoT-based systems for precision irrigation are mostly theoretical with limited proof of concept experiences. They are either too generic or too specific and do not explicitly address easy system deployment for facilitating replicability and streamlining the deployment of new systems. When it comes to providing advanced features to water management, there are some isolated initiatives not necessarily connected to the existing platforms and architectures. For example, the FIGARO project aims at increasing water productivity and improving irrigation practices through the development of a precision irrigation management platform, but not directly involving IoT [9]. Also, Popović et al. [10] present a case study of a specially designed and currently limited IoT-enabled platform for collecting data in precision agriculture and ecological monitoring domains. Agri-IoT [11] is a theoretical IoT-based framework for data analytics and real-time processing for smart farming that shares some similarities with SWAMP.

In the last years, much has been said about the prospective uses for IoT combined with cloud-based services and big data analytics. In Europe, there is a current concern to understand the challenges and compelling impacts of IoT in large-scale pilots for smart agriculture. Brewster et al. discuss the deployment of those large-scale pilots for IoT in agriculture and describe technologies and solutions that might be present in some agrifood domains, such as dairy, fruit, arable crops and meat & vegetable supply chain [12].

FIWARE has been used as a computing platform for many IoT-based applications for smart farming. Rodriguez et al. [13] compiled a short literature review and presented the Agricolus platform for precision farming. López-Riquelme et al. presented an implementation of FIWARE for a specific scenario of precision irrigation in agriculture in the south of Spain [14], however, it is focused on a specific use case, providing details of devices and equipment, as well as irrigation techniques. In contrast, this paper presents an architecture and a platform based on FIWARE, as well as configurations for system deployments in four scenarios.

Fog computing is a fairly new paradigm aimed at dealing with challenges related to the huge amount of data that will be generated with the increasing utilization of IoT-based systems [15]. A new technological trend to implement the fog is container-based virtualization, which provides a lightweight alternative to traditional hypervisors [16]. FIWARE Generic Enablers are also distributed as Docker containers in order to be used in the SWAMP fog computing approach. FogFlow provides a programming model for IoT-based applications for smart cities distributed over the cloud and the fog located in the network edge [17]. Even though FogFlow is integrated into FIWARE, the SWAMP project takes a clean approach and uses directly the components provided by FIWARE, in combination with new components developed specifically for the SWAMP precision agriculture scenarios whenever needed.

### 2.2. The FIWARE Platform

FIWARE [5] has been attracting general attention for being an open source EU-funded solution, comprised of a series of software components called Generic Enablers (GE) that perform functions needed in IoT-based smart applications. GEs can be used to build different applications that exchange information through a REST API following the OMA NGSI [18] standard based on JSON. The central aspect of the FIWARE NGSI Context Management information model is the concept of entities and their attributes. There has been an ongoing effort to develop a Context Information Management API based on recent advances in Linked Data (LD) [19] based on JSON-LD called NGSI-LD, defined in RDF (Resource Description Framework) [20]. Both NGSI and NGSI-LD are currently supported by FIWARE, even though the NGSI-LD specification is preliminary [21].

Some FIWARE GEs are considered key enablers, such as:

- Orion: A publish/subscribe context broker, considered the heart of FIWARE. Orion only stores the latest version of entity attributes and it needs to work together with other applications in order to maintain historical data.
- IoT Agent: Maps data coming from sensors and going to actuators to the FIWARE NGSI information model to be stored in Orion and further processed by other GEs or external applications.
- Quantum Leap: New GE that preserves Orion NGSI historical entity data as times series, replacing the old and less scalable STH Comet.
- Cygnus: A data processing and distribution system for applications that require more elaborate data flow management than simple historical data.
- Cosmos: A set of tools that interface with popular Big Data Platforms.

It has been shown that different architectural choices of IoT platforms affect system scalability and that automatic real-time decision-making is feasible in an environment composed of dozens of thousands of sensors continuously transmitting data [22]. Cruz et al. present a comprehensive study that proposes qualitative and quantitative metrics and evaluates the performance of various IoT platforms [23]. They did not focus on specific features of FIWARE and did not evaluate different scenarios and infrastructures, such as fog computing. Martínez et al. [24] give a detailed description of the architecture of a testbed of the FIWARE platform configured for the precision agriculture domain. It differs from our approach because their test application connects directly to FIWARE using NGSI JSON interface, while this paper uses an IoT Agent for MQTT and an IoT sensor simulator for generating synthetic data.

A preliminary performance analysis of FIWARE for SWAMP revealed that fog computing does not always improve the overall system performance and can even make it worse [25]. This paper differs from previous work as it evaluates different configurations of FIWARE and its focus is on the scenarios of smart irrigation in agriculture defined for the SWAMP project.

### 2.3. The SEPA Platform

The SPARQL Event Processing Architecture (SEPA) [6] enables the detection and communication of data changes for the Semantic Web of Things [26]. SEPA is based upon W3C SPARQL 1.1 [27], a RDF query language, where publishers and subscribers exchange data. The SEPA framework offers developers a solution for implementing Dynamic Linked Data applications and services. Linked Data refers to a set of best practices for publishing and connecting structured data on the Web [18]. Dynamic aspects of Linked Data involve discovery, granularity level, description of changes, detection algorithms, and notification mechanisms. The SEPA broker is the core element of the architecture, implementing a content-based publish-subscribe mechanism where publishers use SPARQL 1.1 Updates to generate events and subscribers use SPARQL 1.1 Queries to subscribe to events. The latter receives SPARQL query results and subsequent notifications generated by changes in the RDF knowledge base are expressed in terms of added and removed query results since the previous notification.

## 3. SWAMP: Concept and Overview

The SWAMP project is developing a high-precision smart irrigation system concept for agriculture. Within SWAMP, water management for agriculture is partitioned into three phases: water reserve, water distribution and water consumption. For Water Consumption SWAMP provides real-time responses for adapting irrigation as crop conditions change. On the other hand, changes in water distribution are performed in a longer timescale. Distribution and Consumption management systems are integrated, as water usage triggers water distribution. The management of water reserves is not considered.

**Figure 1:**
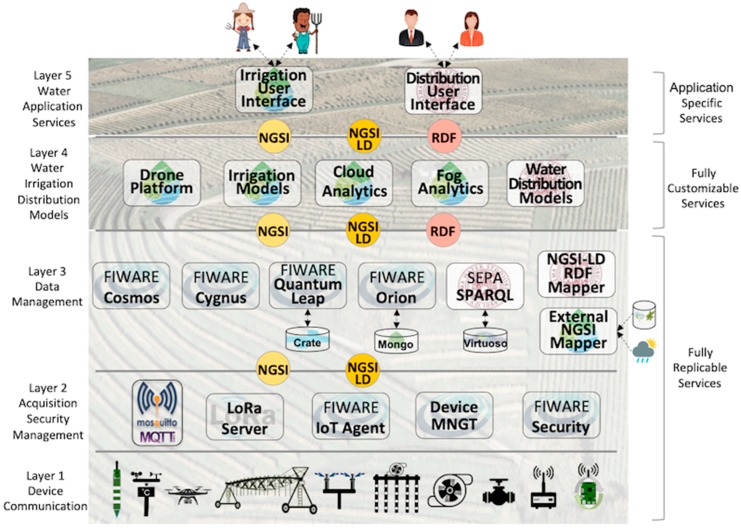
SWAMP Layered Architecture.

The SWAMP Architecture is divided into five layers, as depicted by Figure 1.

- Layer 1: Device & Communication: a variety of sensor and actuator technologies to acquire soil (e.g., moisture), plant (e.g., growing stage) and weather (e.g., air temperature), as well as LPWAN communication technologies (e.g., LoRaWAN [28]) are abstracted in this layer. The SWAMP pilots use commercial sensors as well as a homemade multiparametric sensor probe. Also, commercial drones have been used to take images but a specific drone is under development since one of the partners is a drone maker. A complete description of the sensing infrastructure is outside the scope of this paper.
- Layer 2: Data Acquisition, Security & Management: protocols and software components for data acquisition (e.g., MQTT [29] and LoRa Server [30]) are the key characteristic of this layer, in addition to security and device management functions. The FIWARE IoT Agent GE also belongs to this layer as it translates the internal FIWARE data representation in JSON from/to devices.
- Layer 3: Data Management: contains software components in charge of data storage, processing and distribution based on FIWARE GEs (Orion, QuantumLeap, Cygnus and Cosmos) and SEPA SPARQL engine. A mapper between FIWARE JSON NGSI and SEPA RDF data models also belong to this layer, as well as a mapper from external data sources, such as historical agriculture yield databases and weather forecast services. A distributed infrastructure composed of cloud servers and fog nodes work together for dealing with massive amounts of data and make it available to the upper layers.
- Layer 4: Water Irrigation & Distribution Models: traditional agriculture models for estimating plant water needs using images generated by drones (crop-based approach) and using soil sensors for determining soil moisture (soil-based approach) belong to this layer. Optimization models and techniques for water distribution based on plant water needs are essential whenever collective networks replace individual water sources. Also, computational intelligence (e.g., machine learning) works together with traditional models or in place of them.
- Layer 5: Water Application Services: irrigation services that make sense to farmers and water distributors via user interfaces.

Different layers of the architecture are comprised of more generic components that are more prone to be ported to other settings, whereas others are more application-specific and thus porting requires new development efforts. When it comes to the generality/specificity scale, the SWAMP architecture provides three categories of components: (a) Fully Replicable Services: Layers 1, 2 and 3 of the architecture are generic enough to allow them to be replicable in different settings; (b) Fully Customizable Services: Layer 4 provides services that are closer to the final application and therefore must be aware of a level of detail that may vary for different techniques and models of water distribution and irrigation, which may require customization for every new deployment; (c) Application Specific Services: Services in Layer 5 address particularities of pilots.

## 4. SWAMP Pilots

### 4.1. MATOPIBA Pilot (Luís Eduardo Magalhães—Brazil)

**Figure 2:**
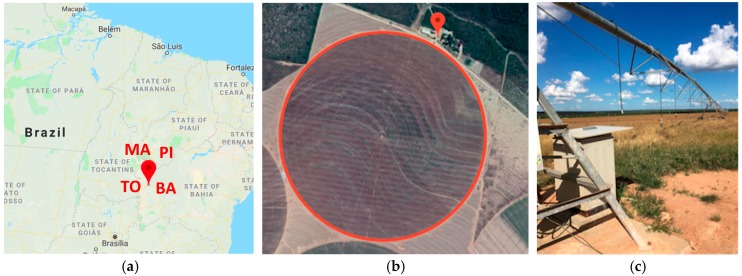
MATOPIBA Pilot: (**a**) Map of Brazil showing the four states where MATOPIBA is located; (**b**) Rio das Pedras farm showing the farm office and the plot; (**c**) Center pivot irrigation in the soybeans plot.

The MATOPIBA region encompasses the Brazilian states of Maranhão (MA), Tocantins (TO), Piauí (PI) and Bahia (BA), and is one of the most critical irrigated agriculture frontiers in the country, located in the cerrado, a savannah climate subtype. Typical crops are soybeans, corn and cotton where irrigation is mostly performed by center pivots. Figure 2 depicts the map of Northeastern Brazil showing the precise location of the pilot in the municipality of Luís Eduardo Magalhães (a), highlights the Rio das Pedras Farm Office and the circular plot (b) that is irrigated by a center pivot (c).

The key challenge for the MATOPIBA pilot is to reduce energy consumption that represents up to 30% of the production cost, by implementing and evaluating a smart irrigation system based on Variable Rate Irrigation (VRI). VRI is able to provide the same yield with about 30% of water usage [31] (up to 50% depending on the soil type), which decreases the cost of energy.

### 4.2. Guaspari Pilot (Espírito Santo do Pinhal—Brazil)

**Figure 3:**
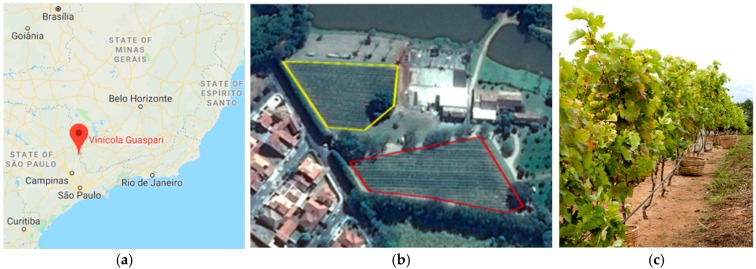
Guaspari Pilot: (**a**) Map of Brazil showing the location of the Winery related to major cities; (**b**) Guaspari winery and the plots where the pilot is located; (**c**) Drip irrigation in the vineyard.

The Guaspari Winery is located in the Brazilian Mantiqueira Mountain Range, municipality of Espírito Santo do Pinhal in the state of São Paulo, where high technology is used to produce high-quality altitude wines. The different terroirs that compose the vineyard are divided into plots, whose altitude ranges vary between 800 and 1300 m. Figure 3 depicts the map of Southeastern Brazil showing the precise location of the pilot (a), highlights the winery and two selected plots (b), and the vineyard featuring the drip irrigation system during the harvest (c).

The key challenge of the Guaspari pilot is to improve the quality of grapes and wines, by performing automatic measuring of soil water content at different soil depths and to provide quick and accurate irrigation management information. The Guaspari Winery uses drip irrigation in its vineyards. By applying a different irrigation map to each vineyard zone, makes it possible to understand the differences of grape quality in different soil types for making better wine blends.

### 4.3. Intercrop Pilot (Cartagena/Spain)

**Figure 4:**
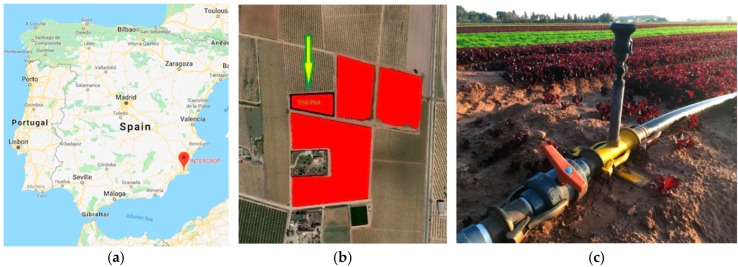
Intercrop Pilot: (**a**) Map of Spain showing the location of Intercrop Iberica; (**b**) Plot where the pilot is located; (**c**) Sprinkler irrigation in a spinach crop.

The Intercrop Pilot is located in Cartagena in the south of Spain within the premises of Intercrop Iberica. Even though Cartagena is located on the coast, it faces a serious water scarcity problem, being located in a semi-arid area with a very short rain season. A considerable amount of water comes from a desalination plant, what contributes to make it a scarce good. These conditions make the case for maintaining internal reservoirs and making a very rational use of water, since there is no guarantee that the supply will meet the demand. Figure 4 depicts the map of Spain showing the precise location of the pilot (a), the plot entirely dedicated to the pilot (b) and the sprinkler irrigation system in a spinach crop (c).

The key challenge of the Intercrop pilot is to reduce the water used in irrigation and to maximize yield per amount of water. In the selected pilot plot two crops are grown in the same season: spinach and lettuce, irrigated by portable sprinkler and drip systems, respectively.

### 4.4. CBEC Pilot (San Michele/Fosdondo—Italy)

**Figure 5:**
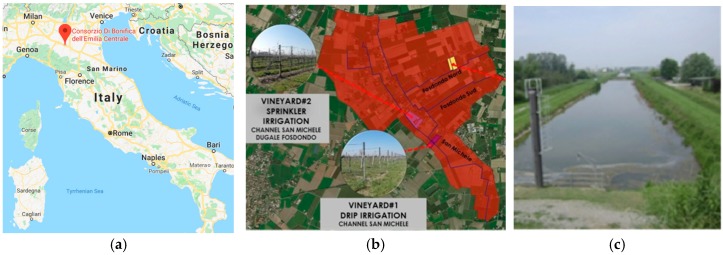
CBEC Pilot: (**a**) Map of Italy showing the location of CBEC office; (**b**) San Michele and Fosdondo areas where the three pilot farms are located; (**c**) Irrigation canal.

The Consorzio di Bonifica Emilia Centrale (CBEC) is a reclamation consortium responsible for irrigation and water drainage in the Emilia-Romagna Region in Northern Italy. The water is distributed to the farms by an intricate infrastructure composed of about 3500 km of canals and dozens of pump stations. The irrigation network consists of open channels on earth, and their filling uses substantial water volumes that frequently are subject to a high loss rate, due to evaporation and infiltration through canal banks and bottom. Figure 5 depicts the map of Italy showing the location of CBEC office in the municipality of Reggio Emilia (a), the San Michele and Fosdondo areas where the three pilot farms are located (b), and an irrigation canal filled with water (c).

The key challenge of the CBEC Pilot is to optimize water distribution to increase usage of water passing along the canals, based on the real irrigation demand coming from three selected farms, which grow different crops (vineyards and pears) and use different irrigation systems. This optimization will allow CBEC to significantly reduce water waste and energy used in pumps by advanced management practices, and to optimize the irrigation by monitoring the water balance at the farm.

## 5. SWAMP System Deployment Scenarios

It is useful to make a clear distinction between the SWAMP Platform and a SWAMP System. The platform is a set of generic components depicted in Figure 1, used to build SWAMP Systems. Whereas there is only one SWAMP Platform, each pilot requires a system to be deployed to fulfill its specific needs of irrigation or water distribution. Generic components, mainly those from the lower layers of the architecture belong to the platform and may be deployed in different ways in any system. On the other hand, specific components such as user interfaces belong to the system.

### 5.1. SWAMP Baseline Scenario

**Figure 6:**
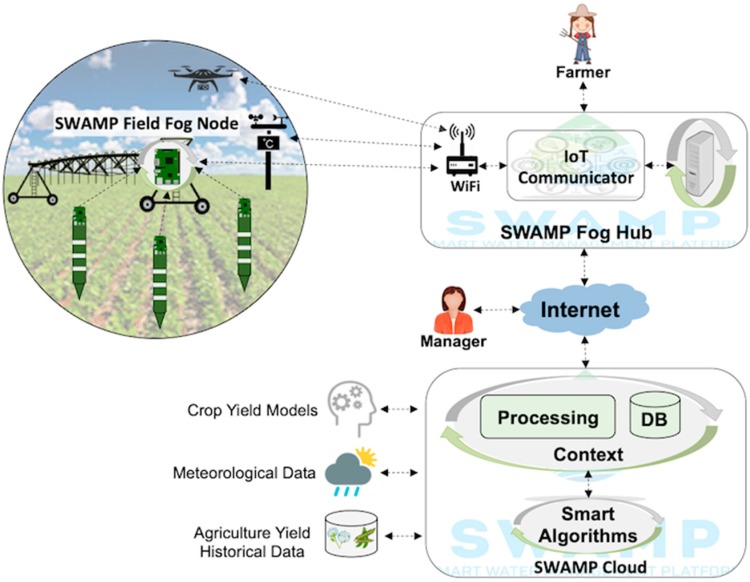
SWAMP Baseline Scenario including SWAMP Cloud and SWAMP Fog (further organized into SWAMP Fog Hub and SWAMP Field Fog Node).

Figure 6 depicts a baseline deployment scenario for the SWAMP platform that includes three key components, which are the SWAMP Cloud, the SWAMP Fog Hub and SWAMP Field Fog Node. This scenario represents the most complete and distributed version of the SWAMP Platform, which is not necessarily deployed as is in the four pilots. For example, a fog infrastructure is not necessary in every system deployment of the SWAMP platform.

Figure 6 also highlights a hierarchical organization of the fog, divided into Fog Field Nodes (FFN) and a Fog Hub in the farm office. A FFN has a stable power source and provides data transmission, storage and processing capabilities to the sensors spread in a farm plot, i.e., behaving as an aggregation point for LPWAN devices [28]. Irrigation systems like the center pivot can host a FFN or even drones. Its implementation may be as simple as a LoRaWAN Gateway that forwards data to the Fog Hub or may include storage and processing.

The role played by the Fog Hub may also vary depending on farmer’s requirements of robustness to Internet disconnections, which may be frequent in agriculture frontiers. The functionalities provided by a Fog Hub may vary from a sensor aggregation point to a full-fledged mini cloud data center if farmers are willing to have full control of their irrigation systems. The SWAMP Cloud stores and processes data and smart algorithms are based on existing agriculture models and on novel computational intelligence techniques, such as machine learning.

### 5.2. SWAMP System for the MATOPIBA Pilot

The deployment of the SWAMP Platform for the MATOPIBA pilot has to deal with communication instability, distance from the farm office to the center pivot, and even distance from the farm to the nearest downtown area A center pivot irrigates a circular agricultural plot of 100 hectares that alternates soybeans and cotton and the plot is further divided into different management zones based on differences in the soil properties. The center pivot controls the variable rate irrigation sprinklers (actuators). The authors developed multiparametric probes for soil sensing, which include moisture, temperature and electrical conductivity sensors at three depths. In intensive agriculture areas the plan is also to experiment ultra-low-power wireless sensor solutions based on wake-up radios combined with LoRaWAN [32].

**Figure 7:**
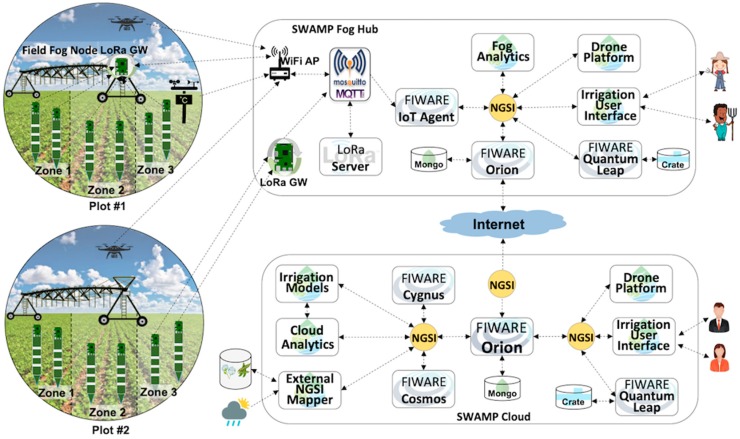
MATOPIBA Scenario represented in the FIWARE-based SWAMP Platform.

Figure 7 depicts the MATOPIBA scenario where cloud and fog software components of the SWAMP Platform are based on the SWAMP architecture (Figure 1). In order to represent different system deployment possibilities, the picture shows two plots, where the topmost plot uses a Field Fog Node (FFN) to aggregate sensor data and the bottommost plot represents the sensors sending data directly to the Fog Hub. Currently, both alternatives are under consideration but only one will be used in the final version of the pilot scenario.

In its simplest possible implementation, the Field Fog Node only contains a LoRaWAN gateway. On the other hand, the complexity of the communication and processing of sensor data is dealt with by the Fog Hub. A LoRaWAN Gateway (located either in the FFN or in the Fog Hub) forwards sensor data via a Mosquitto MQTT Broker [33] to a LoRa Server that in turn sends the data to the FIWARE IoT Agent. The IoT Agent converts the data format into NGSI, which goes to FIWARE Orion Context Broker. FIWARE Quantum Leap is needed for different applications, such as the User Interface and the Drone Platform. Also, a lightweight Fog Analytics component based on simpler statistical techniques is used for computing the irrigation prescription map whenever the Internet connection with the cloud is not available.

The Cloud is represented with its essential components, which include a FIWARE central Context Broker Orion connected (subscribed) to the Fog Orion. Complex processing, such as irrigation models and analytics using smart algorithms (i.e., machine learning), is performed in the cloud. FIWARE Cosmos is used for big data analytics. Traditional models for precision irrigation, such as the FAO Penman-Monteith method [34], are also provided and their results will be used together and compared with machine learning algorithms for determining soil moisture. External information such as crop yield models, weather forecasts and historical data, is fed to the platform by specific NGSI Mappers.

### 5.3. SWAMP System for the Guaspari Pilot

**Figure 8:**
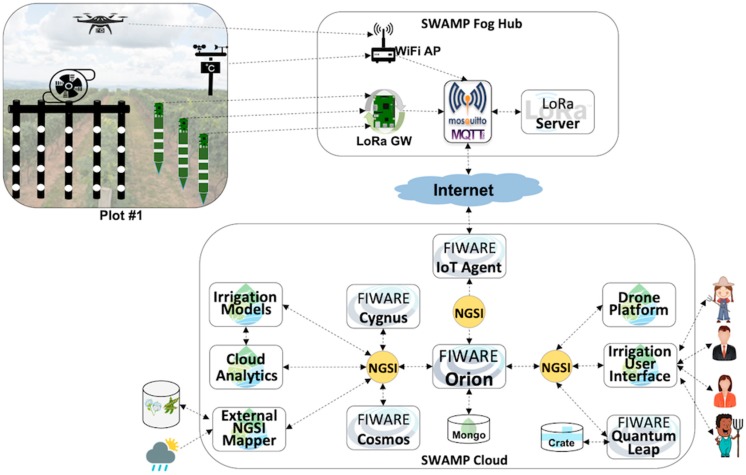
Guaspari Scenario represented in the FIWARE-based SWAMP Platform.

The Guaspari scenario, depicted by Figure 8, is a simplified version of MATOPIBA based on the same SWAMP Architecture, but using a smaller set of components. This system deployment eliminates the Field Fog Node and implements a lightweight version of the Fog Hub, only comprised of communication components, i.e., LoRa Gateway, LoRa Server and Mosquitto. The processing components that calculate the irrigation prescription map, as well as drone-related functions are executed in the cloud. This decision makes it less robust to disconnections but more robust against failures of the fog components.

The IoT Agent is placed in the cloud, where it converts specific data formats coming from sensors and transmits them via wireless communication technologies to store them in Orion. Unlike the MATOPIBA scenario, in Guaspari the data format coming from the field is transmitted over the Internet. Although the IoT Agent plays a communication role and thus could also be placed in the fog, experience has shown that sending verbose NGSI messages over the Internet impairs system performance and brings no noticeable benefits [7].

### 5.4. SWAMP Platform Scenario for the Intercrop Pilot

**Figure 9:**
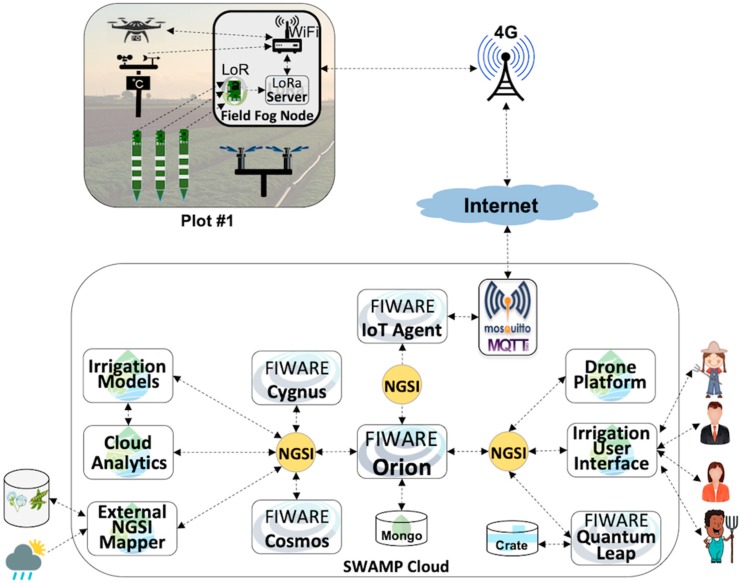
Intercrop Scenario represented in the FIWARE-based SWAMP Platform.

In the Intercrop scenario, depicted by Figure 9, the communication and storage infrastructure is again a simplification of both MATOPIBA and Guaspari scenarios, where no Fog Hub is deployed in the farm office. However, since in this case the sensor data have no route to the Internet (and the cloud), it needs to reintroduce the Field Fog Node (FFN) using LoRa Gateway and LoRa Server for soil sensors and WiFi for drones and weather stations. In this scenario the Mosquitto MQTT Broker is also placed in the cloud, thus becoming the entry/exit point for all incoming and outgoing messages. This configuration of the SWAMP Platform is based on the premise that there is a stable cellular connection (3G/4G) for the FFN to interact with the cloud. In this scenario, apart from the FFN, the farm becomes independent of any IT operations infrastructure for running the SWAMP Platform.

Unlike the other pilot projects, in the Intercrop pilot the plot area is entirely dedicated to the experiments with IoT and irrigation. In other words, it means that the freedom degree is much higher and risks can be taken with less negative consequences. Also, in this pilot the precision level of the irrigation is increased since it deals with smaller and temporary crops, which requires a more detailed monitoring, irrigation and even analytics.

### 5.5. SWAMP Platform Scenario for the CBEC Pilot

The SWAMP Platform Scenario for the CBEC Pilot introduces a significant difference from the previous ones, as it manages both water distribution and irrigation. As such, not only the irrigation prescription map for the farmers must be generated, but also the distribution optimization plan for the water distributor (i.e., CBEC). Also, three different farms participate in the pilot.

Similarly to the Intercrop Scenario (Figure 9), there is no Fog Hub and its functions are handed over to the Field Fog Nodes (FFN) and the Cloud. The FFN has a simpler architecture compared to the Intercrop scenario, where the LoRa Server is placed in the cloud, rather than in the FFN. Additionally, in this scenario FIWARE components are combined with SEPA SPARQL-based semantic engine that represents information in the RDF format and provides contextual semantic queries. In FIWARE the context is established as an entity defined in JSON NGSI format, whereas in SEPA the context is a semantic query. While FIWARE Orion Context Broker notifies all subscribers whenever an entity is changed by a publisher, the SEPA engine notifies subscribers whenever the results of a semantic query is changed, which is more powerful than a single entity.

**Figure 10:**
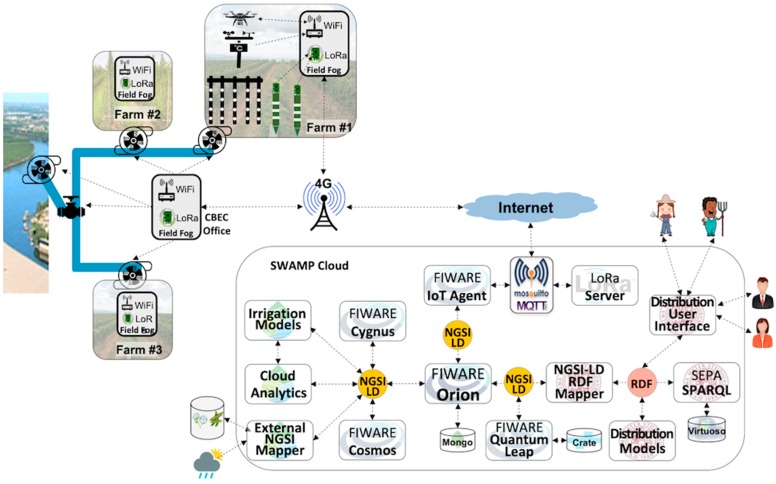
CBEC Scenario represented in the FIWARE-SEPA-based SWAMP Platform.

Figure 10 depicts the FIWARE components already described, along with the SEPA SPARQL Engine, an NGSI-LD/RDF Mapper and two additional applications, the Water Distribution User Interface and the Water Distribution Optimization Models. Orion keeps its role as the data distribution center of the platform but the NGSI-LD (Linked Data) semantic format must be converted to the SEPA RDF format and vice-versa. In this scenario, the FIWARE IoT Agent must be enhanced to generate NGSI-LD.

## 6. Performance Analysis

As the SWAMP Platform is based on FIWARE, the performance and scalability tradeoffs of this solution must be understood. The evaluation of the FIWARE-SEPA integration (Figure 10) requires a different methodology and therefore is not included.

### 6.1. Experimental Design and Research Methods

**Figure 11:**
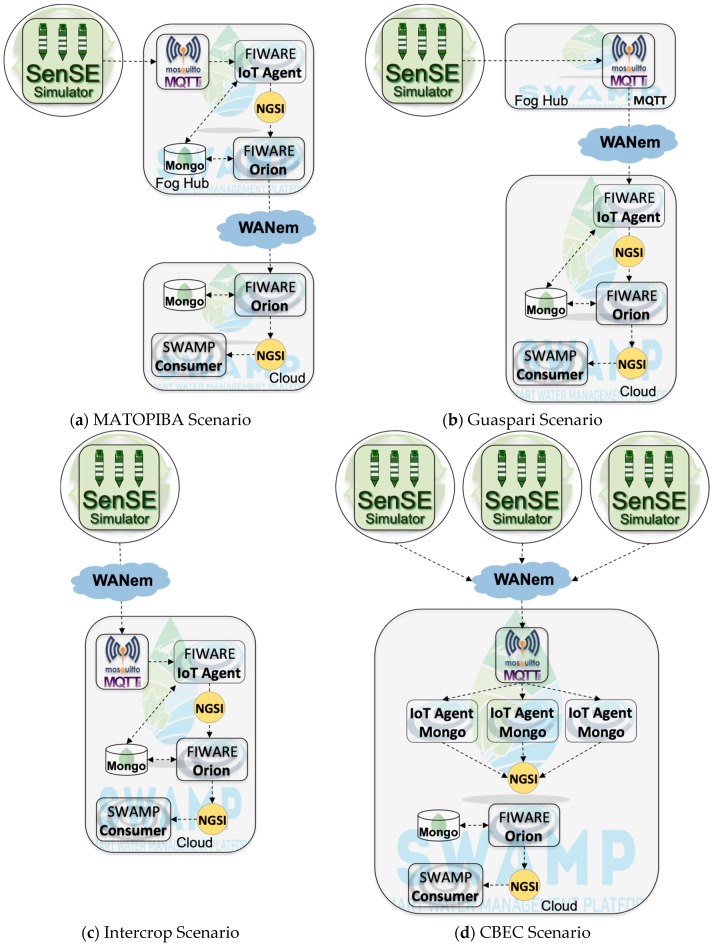
Pilot Scenarios for the FIWARE Performance Analysis of the SWAMP Platform.

In order to analyze the performance and scalability of the four SWAMP pilot scenarios, a FIWARE-based IoT testbed was designed. This involves obtaining sensor data values up to the point where they are transparently consumed by an application that can be deployed in different cloud and fog configurations. Figure 11 depicts the four evaluation scenarios that represent the pilot scenarios introduced in Section 4 abstracted for a testbed evaluation. The key differences between the scenarios are the placement of the components in the cloud or fog. MATOPIBA and Guaspari, Figure 11a,b, are represented as a heavyweight and lightweight fog as in Figure 7 and Figure 8, respectively. Intercrop and CBEC, Figure 11c,d, do not have a fog infrastructure, again because the LoRa components are not evaluated, as in Figure 9 and Figure 10. The CBEC scenario represents the three participant farms, where each one is served by its own IoT Agent in the cloud (and a particular instance of the database), due to performance issues (explained in the results).

The additional components are:

- Sensor Simulating Environment (SenSE): SenSE [35] is an open-source large-scale IoT sensor data generator able to abstract real devices and to model different complex scenarios, such as smart farms [22]. The tool is a traffic workload generator that emulates heterogeneous sensors representing tens of thousands of IoT devices sending data simultaneously via MQTT. Although the sensors are synthetic, the traffic is real;
- Mosquitto MQTT Broker: Eclipse Mosquitto is an open source MQTT message broker;
- MongoDB: a document-oriented NoSQL database, serving as the default Orion storage;
- Consumer: a special purpose web application that subscribes in Orion and receives sensor data from the probes.
- WANem Network Emulator [36]: emulates the Internet connection for the assessment of the impact of network parameters between the place where the data is generated (in the farm,) and the place it is processed (in the cloud).

Regardless of the location of components in the cloud or fog, the sequence of processing steps and data flow is always the same, from source (SenSE) to destination (Consumer); (1) SenSE generates sensor data and sends it to Mosquitto; (2) IoT Agent receives data from Mosquitto, stores it in MongoDB, translates it to NGSI, and sends it to Orion; (3) Orion receives NGSI data from IoT Agent, updates entity values, stores them in MongoDB, and sends them to Consumer; (4) Consumer receives data from Orion and computes the elapsed time since it was generated by SenSE (a timestamp is embedded in the message and physical machines are synchronized by NTP). For the MATOPIBA scenario, there is an additional step, because of the cascading Orion solution, where the Cloud Orion has to subscribe to the fog Orion and the messages are then transferred from one to the other.

A lab testbed emulated the scenario for the experiments. Both fog and cloud were implemented using virtual machines (VM) in OpenStack. The following standard Amazon AWS VM configurations were deployed: cloud VM equivalent to a t2.medium instance (2vCPU—4 GB of RAM) and the fog VMs (both fog field node and fog hub) equivalent to a t2.small instance (1vCPU—2 GB RAM). The cloud was composed of 6 VMs. Two different physical machines were used, with the following configuration: Intel(R) Xeon(R) CPU E3-1240 V2 @ 3.40 GHz—8 cores and 8 GB of RAM.

Three categories of metrics were used in the reported experiments:

- Application metric: The elapsed time is the average time taken since a sensor data point is generated by SenSE until the Consumer receives it. This metric represents how long it takes for sensor data to be available to any subscribed application.
- System metrics: CPU and RAM usage per Docker container, which allows observing each application, collected every 5 s.
- Experiment metrics: The duration of the experiment given by the number of replications (because some components crashed after some time) and the number of received messages are used to understand how experiments unfolded.

The experiments involved a large number of sensors, sending data every 10 min. The scenarios were executed with four different workloads determined by the number of sensors sending messages simultaneously. For the MATOPIBA, Guaspari and Intercrop scenarios, 1000, 5000, 10,000 and 15,000 sensors were used. For the CBEC scenario, as depicted by Figure 11d, the workload was tripled, totaling 3000, 15,000, 30,000 and 45,000 sensors. Each experiment took 1 min and was replicated 30 times, totalizing 16 h of running experiments. Asymptotic confidence intervals were calculated with a 99% confidence level.

The configuration of WANem captured characteristics of a connection from a farm to a cloud based on a simple experiment that obtained the network parameters by pinging a public cloud using a 4G connection, which resulted in a connection of 10 Mbps with 45 ms of delay and 5 ms of jitter.

### 6.2. Results

**Figure 12:**
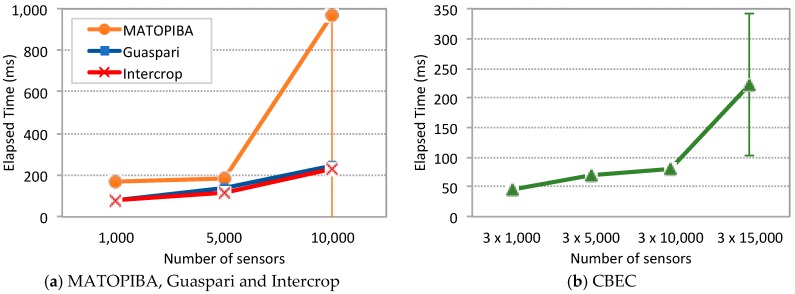
Elapsed time: (**a**) MATOPIBA, Guaspari and Intercrop scenarios; (**b**) CBEC.

Figure 12 depicts the main scalability results according to the elapsed time metric and following the methodology described above. MATOPIBA, Guaspari and Intercrop scenarios are shown in Figure 12a and Table 1 and CBEC scenario in Figure 12b. These results highlight important observations and findings:

- Scalability limits: For MATOPIBA, Guaspari and Intercrop scenarios the viable workload goes up to 10,000 sensors transmitting packets every 10 min (Figure 12a) where an elapsed time less than 1 s was observed. Under a workload of 15,000 sensors, Table 1 shows that the elapsed time increases to dozens of seconds with a high variation.
- Lightweight fog = no fog: The Guaspari and Intercrop scenario yield very similar results for the elapsed time, which reveals that from a performance point of view there is no significant difference if the MQTT Broker is placed in the fog or in the cloud.
- Cascading Orion: The elapsed time is higher for the MATOPIBA compared to the Guaspari and Intercrop, caused by the two cascading Orion components, in the fog and in the cloud, according to Figure 11a. Whenever Orion receives a message it notifies all subscribers of the involved entity. In this scenario, the cloud Orion is a subscriber to the fog Orion, which means that in order for the Subscriber to receive a message, it passes through two Orion notification steps, which is causing the additional delay (up to one second with 10,000 sensors) and larger confidence interval.
- Divide and conquer: The CBEC scenario has three farms represented by three SenSE traffic generators in Figure 11d. In order to scale up to 15,000 sensors per farm (which gives 45,000 sensors), the IoT Agent needed to be replicated in the cloud, each one in a new VM and using a separate MongoDB instance. That solution increased the scalability for the CBEC scenario, which were able not only to deal with 15,000 (which is not viable in the other three scenarios), but also up to 45,000 sensors.

**Figure 13:**
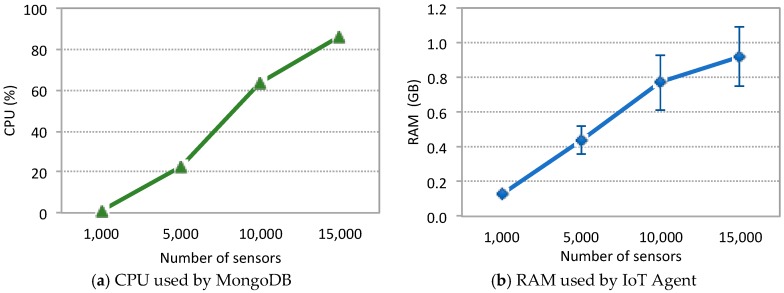
System metrics for the Guaspari Scenario: (**a**) CPU used by MongoDB; (**b**) RAM used by IoT Agent.

The quest for an explanation for the scalability limits of the FIWARE components led us to analyze system and experiment metrics. Figure 13 depicts the percentage of CPU used by MongoDB (a) and the total amount of RAM used by the IoT Agent for the Guaspari scenario (b). The results are equivalent for the other three scenarios. When the system is under a workload of 15,000 sensors the CPU used by MongoDB goes up to 86% (+/−3) and the amount of RAM used by the IoT Agent goes up to 1 GB.

Table 2 also shows that for 15,000 sensors the experiment is finished before all the messages are received (i.e., before 30 min). For example, in the MATOPIBA scenario only 16 replications were performed. Also, it can be observed that the number of messages decreases for 15,000 compared to 10,000, where it is expected to increase.

It was found that the IoT Agent is crashing and causing the experiment to be terminated prematurely, which happens because the IoT Agent needs to allocate memory for the verbose NGSI message whenever it receives a sensor message. There are two possible explanations for that behavior that may be considered either alternative or complementary to each other. On the one hand, it is clearly unreasonable for such a program to grow its memory up to 1 GB (up to 1.4 GB in some cases), which leads us to assume that there is a memory leak. On the other hand, the response time for publishing messages to Orion increases steeply thus increasing the number of simultaneous NGSI messages allocated inside the IoT Agent (which cannot free the memory until it receives a confirmation from Orion). In other words, the increased response time together with a very likely memory leak are causing the IoT Agent to crash.

The analysis of these results leads us to conclude that the key bottleneck of the FIWARE Platform is MongoDB, which is not a surprise whatsoever, since its performance constraints are widely known [37]. In the sequence what happens is that MongoDB delays Orion, which in turn delays IoT Agent, which as the weak link grows its memory up to a point where a crash is unavoidable.

## 7. Discussion

The main purpose of SWAMP is to build an IoT platform for precision irrigation in agriculture focusing on different challenges, such as adaptability, deployment, complexity, and information model:

Adaptability: The platform must be flexible enough to adapt to different scenarios while keeping the human effort at a minimum level. As shown in this paper, the four SWAMP pilots provide enough diversity to assist us in understanding the levels of generality and specificity to be provided by different software components. Initially we identified three levels of components regarding a generality/specificity scale (fully replicable, fully customizable and application-specific) and intuitively placed them in relevant layers of the architecture. However, experience shows new tradeoffs, such as the generality of a component for collecting sensor data, like the FIWARE IoT Agent, which must deal with different combinations of data formats of sensors and wireless technologies.

Deployment: A variety of factors influence the design choices for deployment alternatives of various fog/cloud configuration scenarios: (a) the stability and robustness of the Internet connection in the farm area, which in some cases faces frequent disconnections that must be dealt with, such as in the Brazilian MATOPIBA pilot; (b) the availability of resources and interest of farmers in maintaining an in-house fog-based IoT system in operation with associated service level guarantees similar to a cloud environment, compared to the disruptions caused by cloud intermittent access; (c) the capacity and dependability of the fog nodes; (d) the use of LPWAN technologies for collecting sensor data that might require some minimum farm level infrastructure, such as LoRaWAN, or relying on some yet-to-be-deployed or limited coverage public service, such as NB-IoT or Sigfox [28]. There is no “one size fits all” in IoT systems for precision irrigation, which makes the case for finding different ways of configuring and connecting software components in cloud- and fog-based deployments. The need for an automated mechanism for system deployment, given different requirements, infrastructures and constraints was identified and it is currently handled within the project, but this is out of the scope of this paper.

Scalability: Currently, most open IoT-based systems that report results are proof-of-concept pilots or small-scale IoT-based services. Currently there is no de facto IoT Platform even though there are many candidates, including commercial solutions such as Amazon AWS IoT and Google Cloud IoT. FIWARE was adopted as the underlying IoT platform for the most common functions of data distribution and storage. However, as our performance analysis revealed, FIWARE still needs significant improvements to become a scalable solution for extreme scenarios with heavy data generation. For example, the CBEC consortium provides irrigation water for about 5000 farms and in order for the optimization of water distribution to succeed they will need everything to be dealt with by a single platform. For sure these large scenarios will require a considerable number of devices that will stress the platform even more than our experiments.

Complexity: There is a tradeoff in terms of dealing with complexity when it comes to developing IoT-based applications. The approach taken in the CBEC scenario is based on a hybrid solution that combines a FIWARE-enabled context broker and a semantic engine based on the linked data ontology model. While FIWARE Orion Context Broker notifies all subscribers whenever an entity is changed by a publisher, the SEPA engine notifies subscribers whenever the results of a semantic query is changed, which is more powerful than a single entity. This configuration of the SWAMP Platform allows us to compare a solution where the context broker is simpler and the complexity dwells in the applications (FIWARE) to a solution where the context broker is more complex and thus the applications can be simpler (SEPA).

## 8. Conclusions

The emergence of IoT is a phenomenon that owes to the conjunction of several factors and now starts to become real with huge effort both in research and business areas. In this context, the SWAMP project develops IoT-based methods for smart water management in precision irrigation, and pilots them in Italy, Spain, and Brazil. This paper introduced the SWAMP architecture, pilots and deployment scenarios for the four pilots using FIWARE as the underlying IoT platform.

A performance analysis of key FIWARE components personalized for each SWAMP pilot scenario was undertaken to understand the scalability limits of the system. The results show that this platform might be able to deal with the performance requirements of our pilots, even though requiring specially designed deployment configurations and the re-engineering of some components to provide higher scalability using less computational resources. Particularly, our experiments showed that MongoDB is CPU greedy, which negatively impacts system performance.

SWAMP is an ongoing project and therefore there are multiple paths for future work. Some examples are improving the platform deployment scenarios, reporting the overall working of the SWAMP approach in the pilots, including the experience with irrigation models and analytics and more advanced performance analysis.

## Figures and Tables

**Table 1:** Elapsed time for the MATOPIBA, Guaspari and Intercrop scenarios.

Sensors	MATOPIBA		Guaspari		Intercrop	
	Value (ms)	Conf. Int. (ms)	Value (ms)	Conf. Int. (ms)	Value (ms)	Conf. Int. (ms)
1000	165.7	0.7	79.3	1.0	79.9	1.2
5000	185.1	5.4	136.9	1.8	117.8	2.0
10,000	973.0	1229.8	242.4	18.7	229.3	21.5
15,000	17,417.0	11,270.6	18,979.5	11,216.7	66,549.9	11,648.9

**Table 2:** Experiment Metrics: number of messages received and duration of the experiment (in replications).

Sensors	MATOPIBA		Guaspari		Intercrop		CBEC (Sensors × 3)	
	Rep	Msg	Rep	Msg	Rep	Msg	Rep	Msg
1000	30	2988	30	2992	30	2992	30	8962
5000	30	14,867	30	14,850	30	14,838	30	44,247
10,000	30	29,225	30	29,534	30	29,532	30	42,020
15,000	16	24,826	21	28,811	27	29,212	27	45,647
